# Supplementary material for: The Presence of Mycotoxins in Human Amniotic Fluid
Source: Toxins (Basel). 2021 Jun 9;13(6):409. doi: 10.3390/toxins13060409 (PMC8228883; doi:10.3390/toxins13060409)
Supplement: Supplementary file 1 [file toxins-13-00409-s001.zip › toxins-1219755 supplementary updated.pdf]

Supplementary Materials: The Presence of Mycotoxins in Human Amniotic Fluid

Karolina Gromadzka, Jakub Pankiewicz, Monika Beszterda, Magdalena Paczkowska, Beata Nowakowska and Rafał Kocyłowski

Table S1.

nr	Nr PD	Test Results aCGH	OTA [ng/ml]	AFLs [ng/ml]				NIV [ng/ml]	DON [ng/ml]	ZEA [ng/ml]
				G1	G2	B2	B1			
1	2877	correct	0.5	nd	nd	nd	nd	nd	nd	nd
2	2879	correct	0.2	nd	nd	nd	nd	nd	nd	nd
3	2883	incorrect	0.06	nd	nd	<LOQ	nd	nd	nd	nd
4	2884	correct	0.05	nd	nd	nd	nd	nd	nd	nd
5	2885	correct	0.06	nd	nd	nd	nd	nd	nd	nd
6	2886	correct	0.08	nd	nd	nd	nd	nd	nd	nd
7	2887	correct	0.2	nd	nd	nd	<LOQ	nd	nd	nd
8	2888	incorrect	0.2	nd	1.9	nd	nd	2823.40	nd	nd
9	2889	incorrect	0.7	nd	nd	nd	nd	nd	nd	nd
10	2890	correct	0.3	nd	nd	nd	nd	nd	nd	nd
11	2892	correct	nd	nd	nd	nd	nd	nd	nd	nd
12	2893	correct	0.1	nd	nd	nd	nd	nd	nd	nd
13	2898	correct	0.1	nd	nd	nd	nd	nd	nd	nd
14	2902	correct	<LOQ	0.3	nd	nd	nd	nd	nd	nd
15	2904	incorrect	nd	<LOQ	<LOQ	nd	nd	nd	nd	nd
16	2906	correct	nd	<LOQ	<LOQ	nd	nd	nd	nd	nd
17	2907	incorrect	nd	<LOQ	nd	nd	nd	nd	nd	nd
18	2909	correct	<LOQ	nd	nd	nd	nd	nd	nd	nd
19	2910	correct	nd	nd	nd	nd	nd	nd	nd	nd
20	2911	correct	nd	nd	nd	nd	nd	2099.20	nd	nd
21	2913	correct	<LOQ	nd	nd	nd	nd	nd	nd	nd
22	2918	incorrect	nd	nd	nd	nd	nd	470	nd	nd
23	2920	correct	nd	<LOQ	<LOQ	<LOQ	nd	nd	nd	nd
24	2921	incorrect	nd	nd	nd	0.4	nd	nd	nd	nd
25	2925	correct	nd	nd	nd	nd	nd	nd	nd	nd

26	2928	correct	nd	nd	nd	nd	nd	nd	nd	nd
27	2929	correct	nd	nd	nd	nd	nd	nd	nd	nd
28	2930	correct	nd	<LOQ	nd	nd	nd	nd	nd	nd
29	2931	incorrect	nd	nd	nd	nd	nd	nd	nd	nd
30	2932	correct	nd	nd	nd	nd	nd	nd	nd	nd
31	2933	correct	nd	nd	<LOQ	nd	nd	nd	nd	nd
32	2934	correct	<LOQ	nd	nd	nd	nd	3102.60	nd	nd
33	2936	correct	nd	nd	nd	<LOQ	nd	nd	nd	nd
34	2937	correct	nd	nd	nd	nd	nd	nd	nd	nd
35	2938	correct	nd	nd	nd	nd	nd	nd	nd	nd
36	2939	correct	0.02	nd	nd	nd	nd	nd	nd	nd
37	2940	correct	nd	nd	<LOQ	nd	nd	nd	2417.80	nd
38	2941	correct	nd	<LOQ	<LOQ	<LOQ	nd	nd	nd	nd
39	2943	correct	nd	nd	nd	nd	nd	nd	nd	nd
40	2945	incorrect	nd	nd	nd	nd	nd	nd	nd	nd
41	2946	correct	nd	nd	nd	nd	nd	nd	nd	nd
42	2947	correct	nd	<LOQ	nd	nd	nd	nd	nd	nd
43	2948	correct	nd	nd	nd	nd	nd	nd	nd	nd
44	2949	incorrect	nd	0.3	nd	nd	nd	nd	nd	nd
45	2950	correct	<LOQ	0.2	nd	nd	nd	nd	nd	nd
46	2951	correct	nd	nd	<LOQ	nd	nd	nd	nd	nd
47	2953	correct	nd	nd	nd	<LOQ	nd	nd	613.8	nd
48	2954	correct	nd	0.2	nd	nd	nd	470.2	nd	nd
49	2955	correct	nd	nd	nd	nd	nd	nd	nd	nd
50	2956	correct	nd	nd	nd	nd	nd	nd	nd	nd
51	2959	correct	nd	nd	nd	nd	nd	nd	nd	nd
52	2960	correct	nd	nd	nd	nd	nd	nd	312.6	nd
53	2961	correct	nd	nd	nd	nd	nd	nd	nd	nd
54	2966	correct	<LOQ	nd	<LOQ	nd	nd	nd	nd	nd
55	2994	correct	<LOQ	0.4	nd	nd	nd	nd	nd	nd
56	2995	correct	nd	nd	nd	nd	nd	nd	nd	nd
57	3002	correct	0.03	nd	nd	nd	nd	208	702.4	nd

58	3012	correct	nd	nd	nd	nd	nd	nd	439.5	nd
59	3016	incorrect	nd	nd	nd	nd	nd	285.2	571.4	nd
60	3032	incorrect	nd	nd	nd	nd	nd	nd	1022.80	nd
61	3034	correct	nd	nd	nd	nd	nd	104.1	1034.30	nd
62	3052	incorrect	nd	nd	nd	nd	nd	159	1094.60	nd
63	3073	correct	nd	nd	nd	nd	nd	232.8	2001.80	nd
64	3088	correct	nd	nd	nd	nd	nd	774.3	nd	nd
65	3094	correct	nd	0.02	nd	nd	nd	393.7	332.1	nd
66	3098	correct	nd	nd	nd	nd	nd	329.2	1924.80	nd
67	3110	correct	0.05	nd	nd	nd	nd	136.9	nd	nd
68	3113	correct	nd	nd	nd	0.1	nd	<LOQ	851.8	nd
69	3114	correct	nd	nd	nd	0.2	nd	nd	nd	nd
70	3115	correct	nd	0.1	nd	0.2	nd	201.5	640	nd
71	3118	correct	nd	nd	nd	nd	nd	4037.60	nd	nd
72	3121	correct	nd	nd	nd	nd	nd	835.2	nd	nd
73	3123	incorrect	nd	nd	nd	nd	nd	nd	nd	nd
74	3127	correct	nd	nd	nd	nd	nd	nd	nd	nd
75	3129	correct	nd	nd	nd	nd	nd	242.1	1553.60	nd
76	3190	incorrect	nd	nd	nd	nd	nd	nd	nd	nd
77	3200	correct	nd	nd	nd	nd	nd	477.8	684.3	nd
78	3204	correct	nd	nd	nd	nd	nd	336.9	nd	nd
79	3205	correct	nd	nd	nd	nd	nd	229.9	<LOQ	nd
80	3206	correct	nd	nd	nd	nd	nd	639.7	490.3	nd
81	3218	correct	nd	nd	nd	nd	nd	652.4	523.8	nd
82	3219	correct	nd	nd	nd	nd	nd	1329.00	1592.80	nd
83	3223	correct	nd	nd	nd	nd	nd	645.7	498.8	nd
84	3227	correct	nd	0.1	nd	nd	nd	117.72	993.6	nd
85	3244	correct	nd	nd	nd	nd	nd	572.7	729.1	nd
86	3247	correct	0.03	nd	nd	nd	nd	144.2	544	nd
